# Past Cigarette Smoking Is More Common among Those with Cholinergic Than Noncholinergic Dementias

**DOI:** 10.1155/2014/423602

**Published:** 2014-12-10

**Authors:** Kyle Dalrymple, Erin K. Saito, Natalie Diaz, Julia Morrow, Beau Nakamoto, Aaron M. McMurtray

**Affiliations:** ^1^Pitzer College, Claremont, CA 91711, USA; ^2^Neurology Division, Los Angeles Biomedical Research Institute, Torrance, CA 90502, USA; ^3^Neurology Department, David Geffen School of Medicine at UCLA, Los Angeles, CA 90095-6975, USA; ^4^Neurology Department, Harbor-UCLA Medical Center, Building N-25, 1000 West Carson Street, Torrance, CA 90509, USA; ^5^Neurology Department, Straub Hospital and Clinics, Honolulu, HI 96813, USA; ^6^Neurology Division, Department of Medicine, John A. Burns School of Medicine, University of Hawaii, Honolulu, HI 96813, USA

## Abstract

*Background*. Patients with progressive dementing disorders associated with cortical cholinergic dysfunction gradually develop cholinergic deficits many years before symptom onset and may begin to smoke cigarettes during midlife as a form of self-medication. The aim of this study was to compare self-reported past smoking rates between those with and without cholinergic dementias, to determine if those who developed cholinergic dementias were more likely to smoke during midlife than those who did not. *Methods*. Retrospective cross-sectional study of past smoking status among patients treated at an outpatient clinic during a three-year period. *Results*. A total of 440 patients were evaluated during the study period, including 224 with cholinergic dementias and 216 with noncholinergic dementias and controls. Past smoking rates were greater among those with cholinergic dementias compared to those without cholinergic dementias (43.92% versus 26.96%, *P* = 0.012). Additionally, smokers with cholinergic dementias reported significantly greater mean pack-years of smoking (*P* = 0.038). *Conclusions*. Greater midlife smoking rates and greater pack-years of smoking were associated with cholinergic dementias. These results suggest midlife smoking may be an early indicator for those developing brain cholinergic deficits related to progressive dementing disorders and support initiating treatment prior to symptom onset in cholinergic dementias.

## 1. Introduction

Cholinergic dementias, those associated with progressive degeneration of cortical cholinergic neurons such as Alzheimer's disease (AD), dementia in Parkinson's disease (PDD) vascular dementia, and Lewy body dementia (LBD), show symptoms of cognitive decline related to gradually decreasing levels of brain acetylcholine (ACh) [1, 2]. The cortical cholinergic deficit is especially notable in LBD, which may display the largest relative deficit of cortical cholinergic function out of these disorders [3, 4]. In LBD and other cholinergic dementias, there is an inverse relationship between cortical cholinergic function and disease symptoms, with reduced cortical cholinergic function correlating with worsening cognitive deficits and other symptoms [5]. As cortical ACh levels decrease, there is downregulation of postsynaptic AchRs, resulting in further reduction of cortical ACh levels through decreased reuptake and consequently further worsening of cholinergic system functioning [6, 7].

Cholinesterase inhibitor therapy has long been used in patients with cholinergic dementias as a means of providing symptomatic relief. However, treatment with cholinesterase inhibitors does not prevent neuronal loss and has not demonstrated any disease modifying effect. It is possible that in order for treatment of these disorders to be effective and change the course of the disease it must be started much earlier, before symptomatic onset and before substantial neuronal degeneration has occurred. However, while there have been recent advances with brain amyloid neuroimaging and other potential biomarkers for dementias, there is currently no consensus regarding how to identify potentially at-risk individuals while still young and healthy or the ages at which cortical cholinergic deficits typically begin to occur in these disorders. Consequently, the optimal ages to begin interventions for these disorders are not yet known.

Nicotine has been shown to bind and exert its action at nicotinic AChRs (nAChR) in the brain and is considered a nonselective AChR agonist. Studies have correlated nAChR dysfunction with the neurodegeneration and cognitive deficits of Alzheimer's disease [8]. Functional neuroimaging studies have shown that cigarette smoking results in occupancy and upregulation of nAChR in the human brain [9, 10]. Chronic nicotine treatment has shown protection from nAChR synaptic impairments believed to be induced by A*β* oligomers in rat models of Alzheimer's disease and prevention of impaired learning and short-term memory [11]. Consequently, nicotine obtained from cigarette smoking may be regarded as a form of unknowingly attempted self-medication when used by those developing cholinergic dementias.

Our study was designed to investigate the potential relationship between a history of past cigarette smoking and clinical dementia diagnosis. We hypothesized that those with cholinergic dementias would report greater past smoking rates compared to those with noncholinergic dementias and normal controls. A greater frequency of past cigarette smoking among those with cholinergic dementias compared to those with noncholinergic dementias would add support to the contention that the cortical cholinergic deficit in cholinergic dementias begins many years earlier in life and also add support for earlier intervention before symptom onset.

## 2. Materials and Methods

### 2.1. Participants

This study was determined to be exempt by the local institutional review board since it consisted of a retrospective review of deidentified clinical data. Participants were all adults, over the age of 18 years, who presented sequentially to a community based outpatient neurology subspecialty clinic during a three-year period from January 1, 2010, to December 31, 2013, for evaluation and treatment of cognitive problems, dementia, or Parkinson's disease. All participants underwent a history and physical examination by a board certified neurologist and dementia diagnoses were determined through retrospective analysis of chart notes according to standard clinical diagnostic criteria.

### 2.2. Determination of Cholinergic and Noncholinergic Dementias

For the purposes of this study participants were divided into two groups based on their clinical diagnosis, one for cholinergic dementia present and one for cholinergic dementia absent. The group of participants with cholinergic dementia present included those with diagnoses in which a deficit of brain acetylcholine levels has previously been established as part of the underlying neurochemistry of the disorder, such as Alzheimer's disease, dementia in Parkinson's disease, vascular dementia, and Lewy body dementia [1, 2, 4, 12, 13]. The group with cholinergic dementia absent included diagnoses in which brain cholinergic deficits are not considered a prominent feature of the neurochemistry, such as frontotemporal dementia, Parkinson's disease without dementia, and cognitively normal controls [12].

### 2.3. Determination of Past Smoking Status

A positive history of past cigarette smoking was determined by retrospective chart review of physician outpatient clinic notes. A positive past cigarette smoking status was determined to be present if the patient, caregiver, or family reported the patient smoked cigarettes on a regular basis earlier in life. Pack-year quantifications of past cigarette smoking were recorded when documented in the chart notes.

### 2.4. Statistical Analysis

Participants were divided into two groups for statistical analysis: (1) those with cholinergic dementias and (2) those without cholinergic dementias including those with noncholinergic dementias and cognitively normal controls. Mean values for continuous demographic factors and other continuous variables, including pack-years of smoking, were compared between groups using two-tailed *t*-tests. Nonparametric data, including positive or negative past smoking status, were compared between groups using the chi-square test. All statistical calculations were performed using SPSS version 22.

## 3. Results

A total of 440 patients were evaluated during the study period. One hundred and thirty-six of these patients were excluded from the study because a reliable past smoking history, either positive or negative, was not documented in the chart notes. Past smoking histories were able to be obtained on 304 patients, including 140 with Alzheimer's disease, 16 with dementia with Lewy bodies, 4 with vascular dementia, 21 with frontotemporal dementia, and 29 with dementia in Parkinson's disease, as well as 75 patients with Parkinson's disease without dementia and 19 cognitively normal individuals who presented with memory complaints. Additional information quantifying past smoking amount in pack-years was able to be obtained from 50 of the 114 smokers (43.86%), including 45 of the 83 smokers with cholinergic dementias (54.22%) and 5 of the 25 smokers with noncholinergic dementias and controls (20.00%).

Subjects with cholinergic dementias were significantly older and had a greater mean age of onset compared to those without cholinergic dementias (see Table 1). Those with and without cholinergic dementias did not differ significantly for gender distribution (48.15% and 40.00% female, resp.; see Table 1). A positive history of past cigarette smoking was significantly greater among those with cholinergic dementias compared to those without cholinergic dementias (43.92% versus 26.96%, resp.; *P* = 0.012; see Table 1). Mean pack-years of cigarette smoking were also significantly greater among those with cholinergic dementias compared to those without cholinergic dementias for those participants who had this data recorded in the chart notes (*P* = 0.038; see Table 1).

## 4. Discussion

In this study, we identified an association between presence of cholinergic dementias and a positive history of cigarette smoking, as well as significantly greater pack-years of cigarette smoking among smokers with cholinergic dementias compared to smokers with noncholinergic dementias and normal controls. Because cortical cholinergic deficits are theorized to develop very gradually over several decades prior to symptom onset in these disorders, it is possible that the greater frequency and pack-year history of smoking may indicate a form of unknowingly attempted self-medication used by these patients to treat symptoms from gradually developing brain cholinergic deficits. These findings also suggest that elderly individuals with a positive smoking history who start developing cognitive impairment may be at relatively higher risk for developing cholinergic rather than noncholinergic dementias, although further prospective study in elderly individuals would be needed to determine any predictive value associated with this finding.

The relationship between cigarette smoking, AD, and dementia in general is complicated. In some groups, specifically those with the APOE4 allele, it has been found that smokers have an increased risk for developing dementia and AD [14]. Other reports of studies in the general population have also supported cigarette smoking as an important risk factor for both dementia and AD [15]. Among those with early signs of AD, smokers are known to have greater rates of brain atrophy than nonsmokers, for both generalized atrophy and specifically atrophy of areas related to development of AD such as the hippocampus [16]. However, high levels of lifetime smoking (>50 pack-years) are reported to have an inverse relationship with other degenerative dementias and are associated with a reduced risk for development of LBD and PD [17]. With Parkinson's disease, the inverse relationship is dose dependent and those with the greatest pack-year histories are the least likely to develop these disorders [18].

This study has several limitations. First, the decision to study an outpatient population rather than an inpatient population may have introduced a selection bias, since smoking is a risk factor for nursing home placement [19]. Additionally, because past smoking status and pack-year histories were determined by retrospective chart review of already existing notes, it is possible that smoking histories were actually underreported. Others have shown that smoking histories are frequently underestimated when assessed from patient chart notes, and it is possible that another mechanism for obtaining smoking histories such as an anonymous questionnaire may have provided a more accurate assessment [20]. Also, we were unable to determine the smoking history for many of the patients seen in the clinic because it simply was not documented in the chart notes, raising the possibility of a reporting bias. However, all three of these potential biases would be expected to have affected both groups equally, so we feel that overall the results are relatively reliable. Further study of other community and research center populations using anonymous questionnaires to determine smoking histories would be warranted.

## 5. Conclusions

The results of this study suggest that patients with cholinergic dementias may have greater past cigarette smoking rates than those with noncholinergic dementias and controls. It is possible that the nicotine in cigarettes may act as a form of self-medication for people who are gradually developing a cortical cholinergic deficit. Additional research focusing on the role of nicotinic AchRs in the development and progression of Alzheimer's disease and other cholinergic dementias is indicated. Additionally, future research to identify nicotinic AchR mediated treatments may help to reduce the severity of symptoms patients face as cholinergic dementias progress and may even have the potential for disease modifying effects.

## Figures and Tables

**Table 1:** Comparison of demographic and past smoking history information between those with and without cholinergic dementias.

	Cholinergic dementia present, *n* = 189	Cholinergic dementia absent, *n* = 115	Sig.
Mean age at visit in years	73.17 (S.D. = 12.32)	69.79 (S.D. = 13.26)	**P** = 0.032
Mean onset age in years	60.49 (S.D. = 11.58),	50.96 (S.D. = 13.22)	**P** = 0.004
Gender (% female)	48.15%, *n* = 91	40.00%, *n* = 46	*P* = 0.299
Positive past smoking history	43.92%, *n* = 83	26.96%, *n* = 31	**P** = 0.012
Mean pack-years smoked	52.53 (S.D. = 45.56)	26.80 (S.D. = 18.94)	**P** = 0.038
